# Detecting Defects on Solid Wood Panels Based on an Improved SSD Algorithm

**DOI:** 10.3390/s20185315

**Published:** 2020-09-17

**Authors:** Fenglong Ding, Zilong Zhuang, Ying Liu, Dong Jiang, Xiaoan Yan, Zhengguang Wang

**Affiliations:** College of Mechanical and Electronic Engineering, Nanjing Forestry University, Nanjing 210037, China; dfl@njfu.edu.cn (F.D.); zzl0702@njfu.edu.cn (Z.Z.); jiangdong@njfu.edu.cn (D.J.); yanxiaoan@njfu.edu.cn (X.Y.); nanlinwzg@njfu.edu.cn (Z.W.)

**Keywords:** solid wood panels, defect detection, SSD algorithm, DenseNet network

## Abstract

Wood is widely used in construction, the home, and art applications all over the world because of its good mechanical properties and aesthetic value. However, because the growth and preservation of wood are greatly affected by the environment, it often contains different types of defects that affect its performance and ornamental value. To solve the issues of high labor costs and low efficiency in the detection of wood defects, we used machine vision and deep learning methods in this work. A color charge-coupled device camera was used to collect the surface images of two types of wood from Akagi and Pinus sylvestris trees. A total of 500 images with a size of 200 × 200 pixels containing wood knots, dead knots, and checking defects were obtained. The transfer learning method was used to apply the single-shot multibox detector (SSD), a target detection algorithm and the DenseNet network was introduced to improve the algorithm. The mean average precision for detecting the three types of defects, live knots, dead knots and checking was 96.1%.

## 1. Introduction

**Figure 1:**
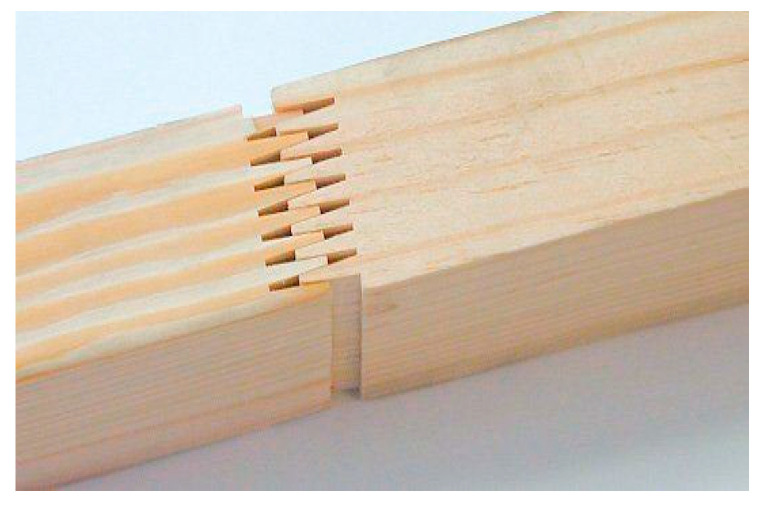
Finger joined lumber.

Wood plays an important role as an essential raw material in many industries, especially in the home and construction industries. However, in China, due to the long growth cycle of most woods, environmental impacts, and resource shortages, production needs are not being met. Additionally Chinese consumers prefer to buy solid wood materials without knots, checkings and wormholes. In order to meet consumer t demand for solid wood panels, Chinese wood processing enterprises are required to spend a lot on labor costs to identify the defects on the surface of solid wood panels, so as to eliminate the defects by sawing and then splice the remaining materials into certain specified plate products through finger joint technology (Figure 1), while also reducing wood waste and increasing the economic benefits. However, the use of manpower to identify the surface defects of solid wood panels has many disadvantages such as strong subjectivity, low work efficiency, high labor intensity, and high cost. Therefore, more and more wood processing enterprises have introduced automation and intelligent wood detection technology to replace humans to identify and detect the quality of the wood, improve work efficiency, reduce costs and increase profits [1].

**Figure 2:**
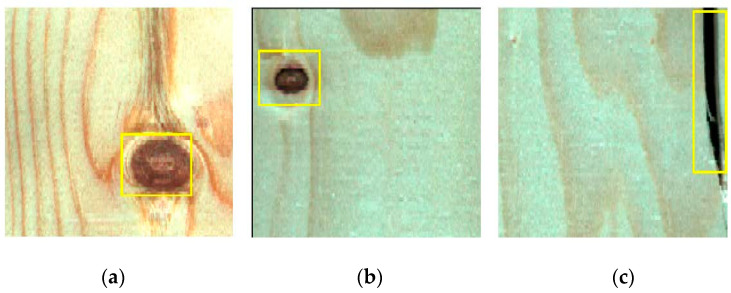
Three types of defect images: live knots, dead knots, and checkings. (**a**) Live knot (**b**) Dead knot (**c**) Checking.

Wood defects refer to various abnormal tissue structures and damage caused by physiological and pathological factors during the growth process of wood or processing. Common defects on the surface of solid wood panels, include live knots, dead knots, checkings and slope of grain [2]. Live knot defects (Figure 2a) are caused when a part of the branches of a living tree are embedded in the main tree trunk. A high number of the live knots complicates the wood pattern and affects the wood’s ornamental value. Dead knot defects (Figure 2b) are caused by dead tree branches. The fiber structure is often partly or completely separated from the surrounding tissues. The existence of dead knots seriously reduces the mechanical properties of solid wood panels. Checkings (Figure 2c) refer to the gaps formed by the separation of wood fibers. Most checkings are caused by external forces. Checkings decrease the wood’s shearing strength parallel to the grain and affect the overall strength of the wood. In addition, fungal infections often occur at checking defects, causing wood to rot and deteriorate. Given that live knots, dead knots, and checkings are the most common defects in the processing of solid wood panels and they have a great impact on the overall quality of the panels, these three defects were considered in this work in order to facilitate the subsequent cutting of solid wood panels.

In the past, contact methods were commonly used to examine or test wood, such as using loading detect the mechanical properties of the wood [3], or using a pin or dielectric moisture meter to detect the moisture content of the wood. In recent years, various methods based on machine vision and computer science have been developed to detect the quality of wood. Non-destructive wood testing methods that are now commonly used include near-infrared spectroscopy testing [4,5,6], ultrasonic testing [7,8,9], X-ray testing [10,11], laser testing [12,13], and acoustic emission technology [14,15,16]. Good results have been obtained by combining the above methods of extracting the surface or internal features of wood with classic machine learning methods, such as back propagation neural network (BP), support vector machine (SVM), and K-means clustering algorithm to predict and classify wood features. With the development of these technologies, wood inspection has gradually made the transition to automated inspection and classification. Due to the ongoing improvement in image acquisition equipment and the expanding role of deep learning technology in the field of image recognition, research has focused on combining machine vision technology with deep learning networks [17] and applying them to the non-destructive inspection of wood surfaces. For example, He et al. [18] used a linear array CCD camera to obtain wood surface images, and proposed a hybrid total convolution neural network (Mix-FCN) for the recognition and location of wood defects; however, the network depth was too deep and required too much calculation. Hu et al. [19], Shi et al. [20] and others used the Mask R-CNN algorithm in wood defect recognition, but they used a combination of multiple feature extraction methods, which resulted in a very complex model. Kurdtongmee [21] introduced the YOLO algorithm for wood pith recognition; however, the study only investigated the detection issues related to wood pith, and did not consider the recognition and multi-classification of wood defects. Indeed, there are numerous non-destructive testing methods for wood and each has its own advantages and disadvantages. Ultrasonic, X-ray, acoustic emission technology, near infrared spectroscopy and other methods are generally used for the detection of internal defect features in wooden structures, but they lack the precision for small target recognition. Compared to the non-destructive wood testing technology mentioned above, machine vision equipment is cheaper than other inspection equipment, and is often used to obtain the surface features of objects. Also, deep learning has shown surprising results, for example, it significantly improves the precision and speed of detection. Therefore, the combination of machine vision and deep learning has become a mainstream method for wood surface inspection.

The purpose of wood defect identification and classification is different depending on the industry. For example, in the construction industry, identifying defects is used to determine the strength characteristics of wood [22]. For us, the purpose of identifying defects is to remove them from the original panels, and then use the remaining high-quality wood to splice into panels to make furniture. The production line in a cooperative enterprise requires a recognition accuracy of higher than 95% for three kinds of defects, namely, live knots, dead knots and cracks on the surface of solid wood panels, and the speed of the conveyor belt should be 50 m per minute. Since the length of each board is 1 m, the scanning time of each board is 1.2 s, so the calculation time for each board image must be less than 1.2 s to ensure that the real-time collection of board images can be realized for the production line. To improve the detection speed and precision of wood surface defects, and meet the requirements of the production line in wood processing enterprises, it is necessary to obtain deeper features of the wood surface image for the identification and location of defects. However, the increase in the depth of the convolutional neural network can cause problems such as an increase in error and gradient disappearance [23]. 

The main innovations of this paper are: (1) the DenseNet network is introduced to extract the deep features in wood images, which avoids the problems of gradient disappearance and increased error, which are often caused by the network being too deep; (2) the feature fusion method in the classic target detection algorithm, the single-shot multibox detector (SSD) was used to fuse the multi-layer feature map obtained by the DenseNet network for the regression of the position parameter of the wood defect in the image and the classification of the defect; and (3) by changing the network parameters, the SSD algorithm was adapted to the 200 × 200 pixel image, and one layer of feature maps was reduced when multi-layer feature maps were merged. These two measures reduced the amount of network calculation and the calculation time. The main contribution of the paper is that our scheme increased the average precision of defect recognition on wood board surfaces to 96%, and the detection time was only about 56 ms, which greatly improves the efficiency of wood surface detection.

## 2. Materials and Methods 

### 2.1. Wood Surface Defect Dataset

**Figure 3:**
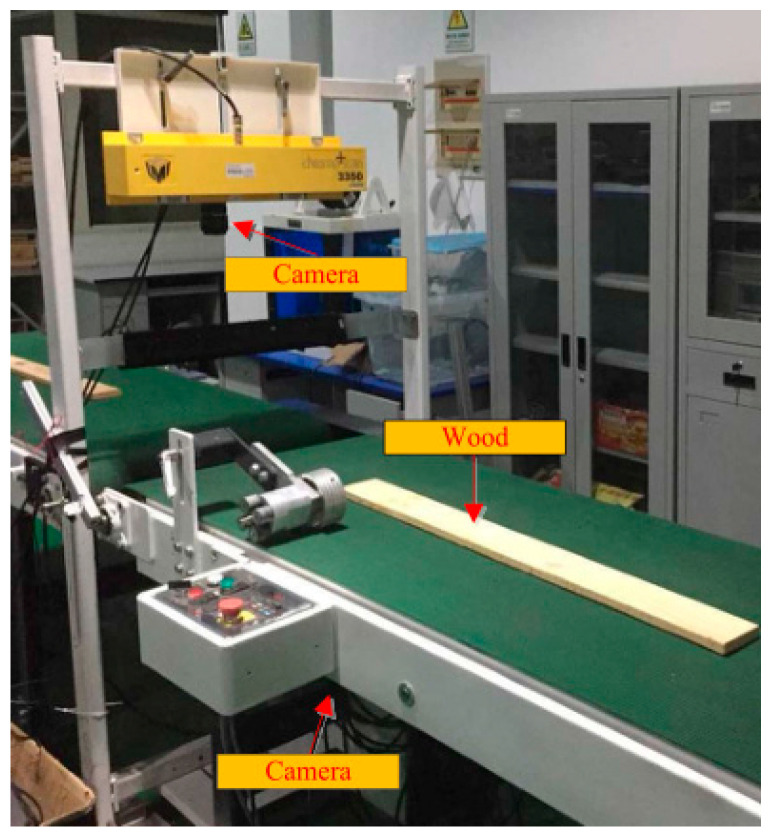
Solid wood board image acquisition equipment.

**Figure 4:**
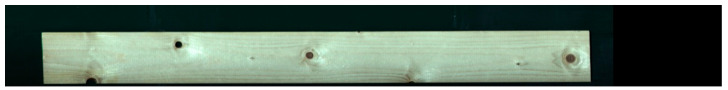
An original image of a solid wood board.

**Figure 5:**
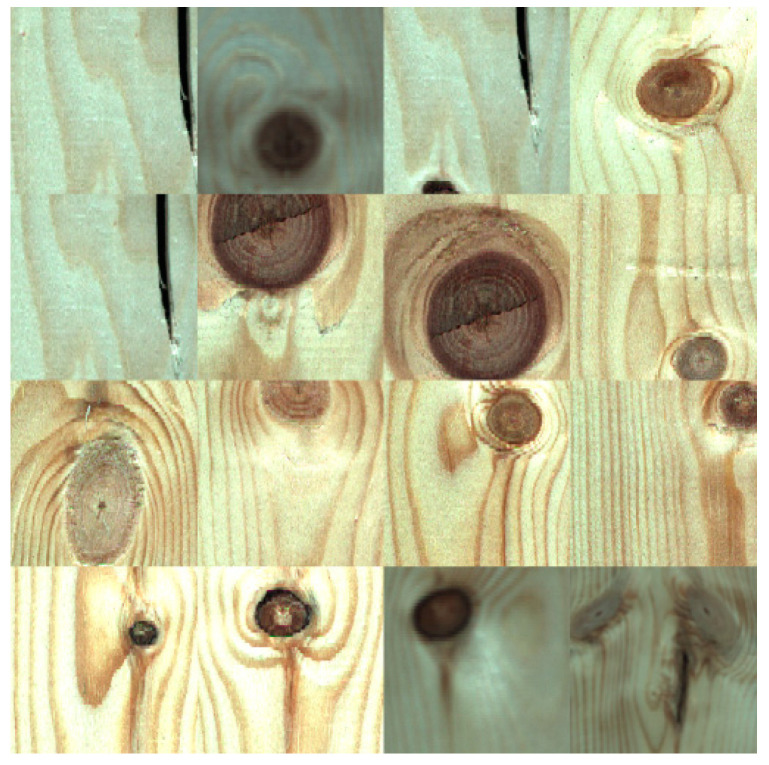
Part of an image containing defects segmented from the original image.

The wood image was obtained by using the self-developed solid wood panel image acquisition equipment shown in Figure 3. The image acquisition equipment consists of two sections of conveyor belt, industrial cameras distributed on both sides of the conveyor belt, a strip light source and a photoelectric switch to trigger the camera. The industrial camera is a DALSA LA-GC-02K05B, the ES12 D15NK photoelectric sensor is produced by LanHon in Shanghai, China, and its detection distance is up to 15 cm. When the solid wood panel moves forward on the conveyor belt, the infrared sensor triggers the linear array CCD cameras distributed on the top and bottom of the conveyor belt to collect double-sided images (Figure 4). In this study, a total of 200 solid wood panels were scanned using two types of wood, Akagi and Pinus sylvestris, and 400 original images of the panels were obtained. The pixels of the original image are 2048 × 18,000, and only a small part of the whole image was defective. Using the convolutional neural network on very large images, will increase the amount of calculation of, use too much memory, and seriously affect the running speed of the network. So, a relatively small image containing wood surface defects was segmented from the original image for network learning. After processing, the collected original image was divided into 200 × 200 pixel images containing different defects (Figure 5), and a total of approximately 500 images were obtained as the original data set.

**Figure 6:**
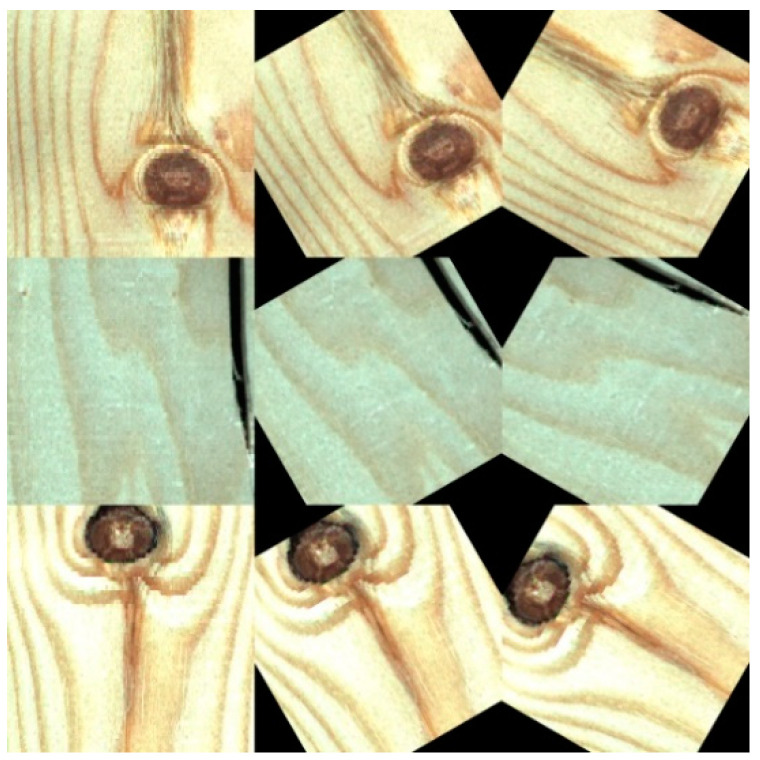
Part of the image generated by the expansion.

In supervised machine learning, small batch data are often divided into a training set and a test set at a ratio of 7:3. The function of the training set is to help us train the model, that is, let us determine the parameters of the fitting curve through the data from the training set. The test set is to test the precision of the trained model. In order to better fit our model, we randomly selected 70% of the original data set as the training set. Then the original training set was expanded to three times the original through four expansion methods. The first method was used to mirror the upper and lower parts of all images in the training set with the horizontal axis of the image as the symmetry axis; the second method randomly extracted one third of the original training set and increased its uniform noise; the third method randomly extracted one third of the original data set and rotated it clockwise by 30 degrees; the fourth method randomly extracted one third of the original data set and rotated it clockwise by 60 degrees. The expanded part of the training images is shown in Figure 6. The remaining 30% of the original data set was used as the test set of the model. After dividing the data and image expansion, the distribution of the samples was as shown in Table 1.

### 2.2. Original Network

#### 2.2.1. Network Backbone

Before deep learning is applied to target detection, the detection of objects by traditional algorithms is commonly divided into three stages: region selection, feature extraction, and feature classification. Traditional algorithms usually use the sliding window algorithm for region selection, perform feature extraction by carefully designing feature extractors, such as SIFT and HOG, and then use classic classification networks, such as SVM and AdaBoost, to classify the extracted features. However, the sliding window algorithm generates many redundant frames and has high computational complexity. The artificially designed extractor contains fewer parameters and is less robust, and the quality of extraction is low.

Convolutional neural networks (CNNs) are currently widely used in image classification and recognition, target detection, and other fields because they can achieve good image feature extraction. Many parameters of deep neural networks can extract features with better robustness and semantics, and their classification performance is also superior. The emergence of regions with CNN features (R-CNN) [24] paved the way for the use of deep learning for target detection. On the basis of R-CNN, Fast R-CNN [25] realizes end-to-end object detection and convolutional sharing and Faster R-CNN provides the anchor mechanism [26]. These networks first focus on finding the location of objects, obtain suggested boxes, and then classify the suggested boxes. This type of algorithm is known as a two-stage algorithm. The other type is called a one-stage algorithm. One-stage algorithms usually rely on the network experience of feature fusion to complete the prediction of the object position and category in one stage. The SSD [27] algorithm used in this study is a classic first-order algorithm. SSD draws on the anchor mechanism in Faster R-CNN and the regression process of the YOLO [28] algorithm. The algorithm improves the speed and detection precision.

**Figure 7:**
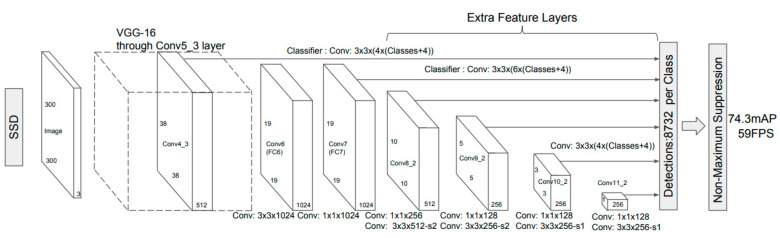
Framework of the single-shot multibox detector (SSD) algorithm.

Figure 7 shows the structure of the framework of the SSD algorithm, which is mainly divided into two parts. One part is the deep convolutional neural network at the front end, which uses the image classification of the VGGNet-16 network (i.e., a 16-layer structure VGGNet) with the classification layer removed and is used for the preliminary feature extraction of the target. The other part is the multi-scale feature detection network at the back end, which is a group of cascaded CNNs that extracts features under different scale conditions from the feature layer generated by the front-end network. The SSD algorithm cancels the fully connected layer in YOLO and maps multi-scale feature maps on the detection layer to adapt to the objective facts of the different target sizes of the input image. After the detection layer, the SSD algorithm uses non-maximum suppression [29] to obtain the maximum value in the local range.

**Figure 8:**
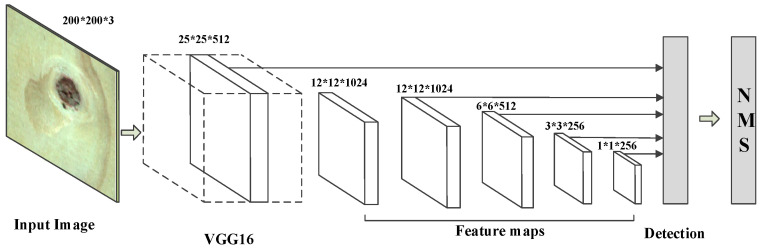
Modified SSD structure.

The SSD algorithm has been widely used in the field of target detection, but its input image is generally 300 × 300 pixels, and our data set was 200 × 200 pixels. In order to ensure the normal operation of the SSD algorithm on our data, we deleted a layer of the feature map on the basis of the original algorithm, and fused five layers feature map for detection and recognition. The modified network structure is shown in Figure 8.

#### 2.2.2. Verifying the Original Network

For the solid wood panel image sample, the image size was first enlarged to 300 × 300 for importing into the SSD model. Although the data set was expanded, the final amount of label data remained extremely small, thus we decided to adopt the transfer learning method, that is, we used the solid wood panel data set to train the pre-trained model on the ImageNet data set. The software, hardware, and environment configurations are shown in Table 2.

**Figure 9:**
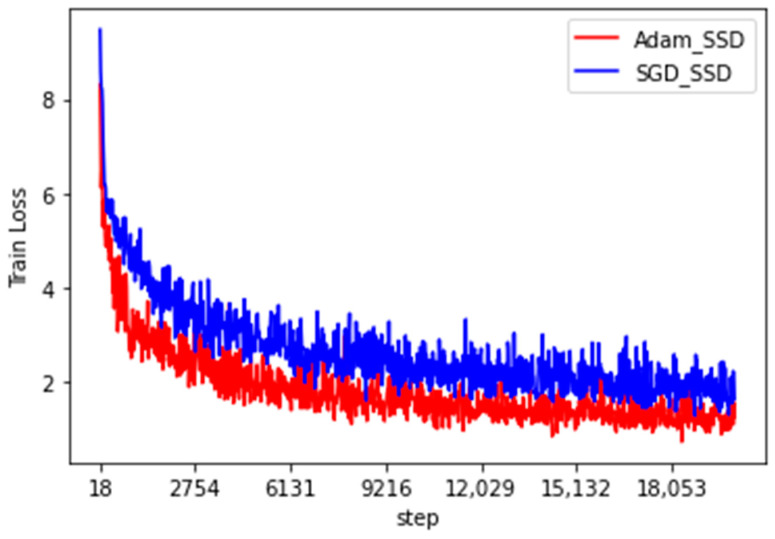
Loss curve of the traditional SSD algorithm under stochastic gradient descent (SGD) and the Adam optimizer in solid wood board defect recognition.

To find the best detection model in iterations, the loss function is used to measure the gap between the predicted value of the object and the true value, and the optimizer is used to gradually reduce the loss value and to finally stabilize it at a lower level, which indicates that the detection model convergence is effective. In the target detection field, the loss function is composed of the positioning loss of the object and the classification loss. The formula is as follows:

(1) $$L\left(x,c,l,g\right)=\;\frac{1}{N}\left(L_{conf}\left(x,c\right)+\propto L_{loc}\left(x,l,g\right)\right)$$

where $L_{loc}\left(x,l,g\right)$ is the object positioning loss. To enhance the robustness of the loss function to outliers, the Smooth L1 Loss function was used to calculate the location loss of defect detection, namely,

(2) $$L_{loc}\left(x,l,g\right)=\;\overset{N}{\underset{i\in Pos}{\sum }}\underset{m\in \left\{cx,cy,w,h\right\}}{\sum }x_{ij}^{k}smooth_{L1}\left(l_{i}^{m}-\;{\hat{g}}_{j}^{m}\right)$$

where $L_{conf}\left(x,c\right)$ is the object classification loss. Commonly used classification loss functions include the mean square error and cross entropy loss functions. The parameter gradient will increase with the loss value. However, when using the mean square error loss function, if the loss value is large, then the gradient of the parameter will decrease instead, but the cross entropy error will not cause this problem, and the loss value can be quickly converged. Therefore, the cross entropy function was used to calculate the classification loss of defect detection, namely,

(3) $$L_{conf}\left(x,c\right)=\;-\overset{N}{\underset{i\in Pos}{\sum }}x_{ij}^{p}\log\left({\hat{c}}_{i}^{p}\right)-\underset{i\in Neg}{\sum }\log\left({\hat{c}}_{i}^{0}\right)\;where\;{\hat{c}}_{i}^{p}=\frac{\exp\left({\hat{c}}_{i}^{p}\right)}{\sum_{p}\exp\left({\hat{c}}_{i}^{p}\right)}$$

where x is the true value of the object, c is the object prediction category, l represents the predicted boxes, g denotes the true boxes, and coefficient ∝ is used to balance the optimized ratio of the two losses and was assigned a value of 1 in this study. The Pytorch framework was used to write the SSD model, and the solid wood panel data set was used for the pre-training model. The number of training iterations was set to 20,000, the learning rate was set to 1 × 10^−4^, and the batch size was set to 16. To find a better loss function optimizer, stochastic gradient descent (SGD) and the Adam optimizer were used for optimization. The graph of the reduction in the training loss value is shown in Figure 9, and the recognition effect of the statistical test set is shown in Table 3.

Figure 9 and Table 3 indicate that the Adam optimizer reduced the loss value of the traditional SSD algorithm faster than SGD and the training loss was reduced from 8.6 to approximately 0.9. The trained model can detect images faster than the SGD method, and the average detection time for each image was only 17 ms. However, the SSD algorithm is generally not effective in identifying the surface defects of solid wood panels. The average precision (AP) was approximately 0.90 for the three types of defects, namely, live knots, dead knots, and checkings. The analysis indicates that the depth of the VGG16 network is insufficient to obtain higher-level image semantic information and extreme loss of information can easily occur during multiple convolution pooling. Therefore, this study optimized and improved the basic network skeleton of the traditional SSD algorithm.

### 2.3. Network Improvement Method

The VGG16 network skeleton is used in the traditional SSD algorithm. Table 4 shows that the VGGNet uses five sets of convolutions and three fully connected layers. The size of the convolution kernel used is basically 3 × 3, and many stacked two 3 × 3 convolution kernels are used. From the perspective of the receptive field, the effect of the same 5 × 5 convolution kernel is the same, but the parameter is smaller and two layers of convolution require two activation functions, which greatly improves the learning ability of convolutional networks. However, when the VGG network reaches a certain depth, increasing the number of layers cannot improve its performance and gradient disappearance and explosion can occur, which affects the convergence of the network and reduces the detection precision.

**Figure 10:**
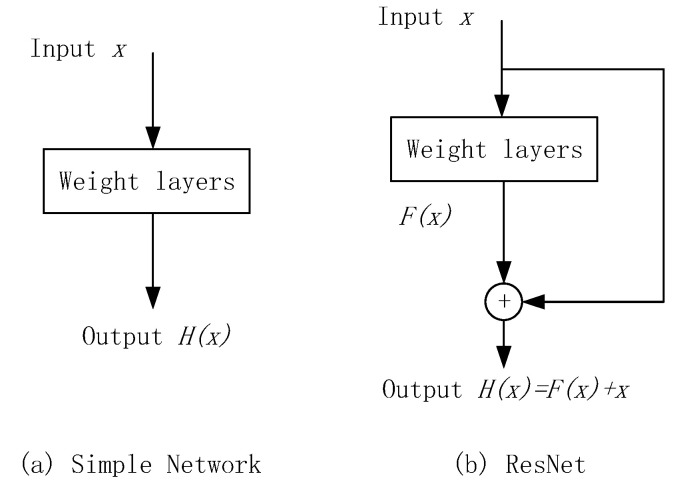
Residual learning: a building block.

To solve this problem, He et al., proposed a deep residual convolutional network (ResNet), which refers to the concept of residual learning (Figure 10), which adds several shortcuts to the feed forward connection to make the network fit the residual mapping instead of the direct mapping from the previous layer. If the desired network is finally mapped to H(x), the network on the left side must be fitted directly to H(x), and the sub-module of ResNet on the right side changes the mapping that must be fitted into F(x)=H(x)−x, by introducing a shortcut branch. ResNet is based on the assumption that optimizing the residual network mapping F(x) is easier than directly optimizing potential mapping H(x). This structure resolves the problem of network degradation caused by the increase in the number of convolutional network layers.

**Figure 11:**
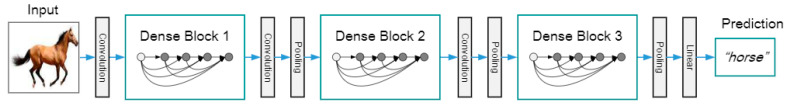
DenseNet network structure.

After ResNet introduced the concept of residual learning into the neural network, Huang et al. proposed a new dense connection convolution network (DenseNet) [30], whereby the information exchange between the front and back layers was maximized. By establishing dense connections between all the front and back layers, feature multiplexing was realized in the channel dimension, which performs better than ResNet with fewer parameters and calculations. Figure 11 presents a diagram of a DenseNet structure containing three dense blocks. The figure shows that in the dense block, the input of each layer is composed of the output of all previous convolutional layers.

**Figure 12:**
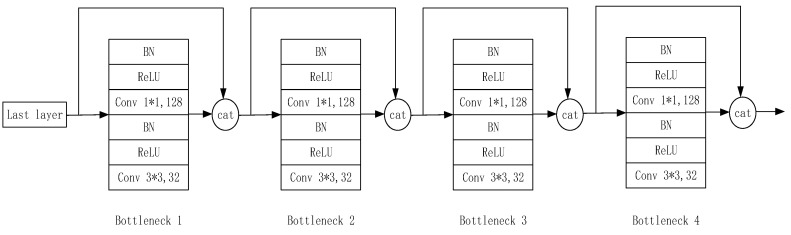
Dense block structure.

The implementation details of the dense block are shown in Figure 12. Each block is composed of several bottlenecks, and each bottleneck is composed of BN, ReLU, 1 × 1 convolution, BN, ReLU, and 3 × 3 convolution in sequence. Then, the channel is concatenated with the result of the previous layer. DenseNet is generally composed of 4 blocks, and the depth of the network is determined by the number of bottlenecks in each block.

To avoid further information loss and prevent the gradient from disappearing, the authors removed the classification layer (Figure 10) of DenseNet, and added a 1 × 1 convolutional layer. Then, this improved DenseNet network replaced VGG16 to extract the image features of solid wood panels and optimized and improved the application of the SSD algorithm in the recognition of solid wood panel defects. Table 5 shows the network structure of DenseNet121, which was used in solid wood board defect recognition.

**Figure 13:**
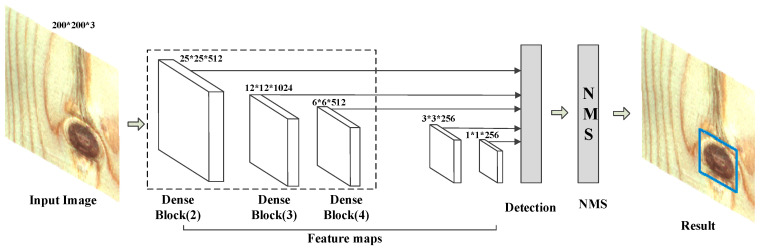
DenseNet-SSD algorithm structure in solid wood panel defect detection.

In the traditional SSD algorithm, the object is detected by fusing the conv4_3 layer of VGG16, replacing the conv_7 layer of the fully connected layer, and adding the feature map obtained by the four convolutional layers. Then, the detection result is obtained through the non-maximum value suppression algorithm. In the improved DenseNet-SSD algorithm, the feature maps were obtained by fusing the Dense Block (2), Dense Block (3), and Dense Block (4) of the DenseNet121 network and using the three newly added convolutional layers as input to the detection layer to better integrate the high-level features with the low-level features and satisfy the feature map size requirements of the feature mapping layer. The specific structure is shown in Figure 13.

## 3. Experiment and Results

### 3.1. Model Performance Indicators

The IoU value, the precision-recall (P-R) curve and the AP value are commonly used as performance evaluation indicators in the field of target detection. The value of IoU refers to the ratio of the intersection between the prediction box and the real box and their union. When the value of IoU is greater than the threshold we set, the prediction box is considered correct, otherwise the prediction is wrong. The horizontal axis of the P-R curve is the recall rate and the vertical axis is the precision rate. Alive knot defect was taken as an example. The recall rate refers to the proportion of all the images with the actual value of a live knot that are detected as live knot defects. Precision refers to the proportion of images whose true value is a live knot among all the images detected as live knot defects. The AP value is the area enclosed by the P-R curve and the coordinate axis, and the value is between 0 and 1. Ideally, the higher the recall and precision rates of each category, the better. The two rates should both be high to avoid reducing the recall rate to increase the precision rate and vice versa. The steeper the P-R curve is, the better, and the figure enclosed by the coordinate axis tends to be a square.

### 3.2. Experimental Results

**Figure 14:**
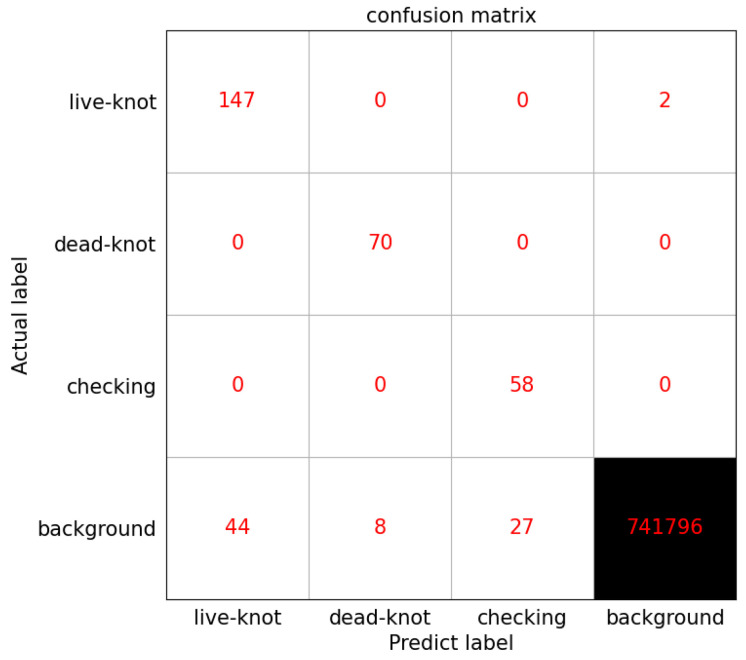
Confusion matrix of prediction box classification results.

By using the same method of transfer learning, the Densenet121’s pre-training model was used on the ImageNet data set to train the data. The batch size value was 16, the learning rate was 1e-4 and the IoU threshold was set to 0.5. During the test, 3638 prediction boxes were generated on each picture, and the number of pictures in the test set was 204, so a total of 742,152 prediction boxes were generated. Since there were a total of 277 defect labels in the test set, most of the prediction boxes were background types. This can also be seen from the confusion matrix in Figure 14. The numbers on the diagonal are the number of correct predictions for each type of defect. The specific test results are shown in Table 6, and the P-R curve is shown in Figure 13. By comparing Table 3 and Table 6, it can be concluded that the SSD algorithm improved by DenseNet significantly improved the overall defect recognition precision, and the detection recall rate of various defects were up to 100%.

**Figure 15:**
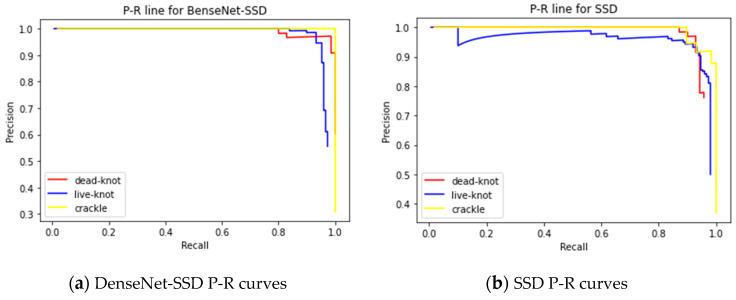
P-R curve of the DenseNet-SSD and SSD algorithm on the solid wood board defect detection test set.

**Figure 16:**
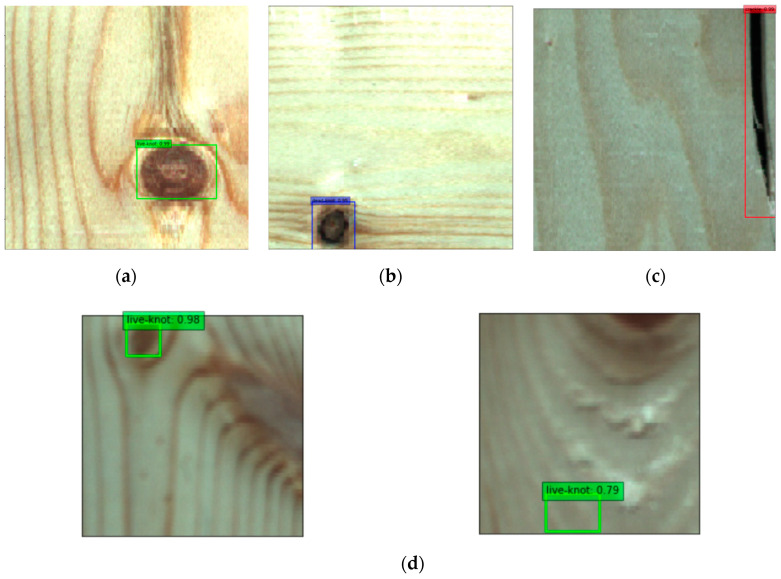
Test results. (**a**) live knot (**b**) dead knot (**c**) checking (**d**) misjudged image.

It can be seen from the confusion matrix in Figure 14 that there are two live knot defect labels predicted as background, and a few of them are mistaken as defects in the prediction box with the true value of the background class. In Figure 15a, the red curve represents the P-R curve of dead knot defects, the blue curve represents the P-R curve of live knot defects, and the yellow curve represents the P-R curve of the checking defects. The P-R curve of the checkings defects is the steepest. When the recall rate of the horizontal axis approaches 1, the precision of the vertical axis can still be maintained at 1. A comparison of the P-R curves of the SSD algorithm improved by DenseNet (Figure 15a) and the traditional SSD algorithm (Figure 15b) indicates that the P-R curve of all types of defects in the former is steeper than that of the latter. Thus, the AP value of the area under the curve is also larger than that of the latter, and the average detection precision of the three types of defects is 0.961. The P-R curve of the live knot defect is taken as an example. When the traditional SSD algorithm is used for detection, the precision rate drops sharply when the recall rate is approximately 0.6, whereas the precision rate decreases after the recall rate reaches 0.8 when the DenseNet-SSD algorithm is used for detection. This finding shows that the DenseNet-SSD algorithm achieves a better balance between the recall and precision rates of various types of defect detection than the traditional SSD algorithm. Therefore, the performance of the improved SSD algorithm is superior to that of the traditional SSD algorithm in identifying solid wood board defects. The detection results for several images are shown in Figure 16. It can be seen from Figure 16d that the reason why the live knot defect was mistaken as the background may be that the texture around the misjudged defect was too complex and was closely connected to the background. The reason why the background was misjudged as a defect may be because the texture and color in the prediction boxes were evenly distributed in the same way as live knots.

## 4. Discussion

In recent years, many algorithms have been developed for the recognition and location of defects in wood surface images with different precision and speed. Although the implemented algorithms are different, the basic processing flow is similar and can be summarized in three categories.

In the first category, the defective image is first extracted from the original image using the image segmentation method, and then different feature extraction methods are used to extract the texture, edge and other types of features of the defect. Then these features are classified by classifiers such as the back propagation (BP) neural network and SVM. For example, mathematical morphology was used to locate the defect location [6], and the spectral information for the defect at different wavelengths was obtained, then the principal component vector was extracted as the input of the BP neural network through principal component analysis. Kamal [31] used the gray level co-occurrence matrix (GLCM) and the law of texture energy measurement to extract texture features of wood images as the input of the BP neural network.

The second category of processing methods appeared with the advent of convolutional neural networks, which are used to extract the features of the entire image, and then machine learning methods are used to perform regression classification on the feature map. For examples, the Faster R-CNN algorithm used by Xiao [32] belongs to this category. It uses CNN as the feature extractor, the region proposal network (RPN) to generate target candidate frames, and the softmax classifier to determine whether there are defects in the candidate frame and the category of the defect. Augustas [33] used the transfer learning AlexNet, VGG16 and ResNet152 networks in Faster R-CNN. A comparison of these methods found that the detection accuracy of wood defects was highest at 80.6% when the RestNet pre-training model was used. Shi [20] designed a glance network at the front end of Mask R-CNN to classify normal panels and panel images with defects, and then input the images of panels with defects into the Mask R-CNN model. The detection accuracy increased to 95.31%. However, because it is a combined model, the subsequent classification accuracy of the Mask R-CNN model is very dependent on the performance of the glance network.

The third category involves the use of fully convolutional neural networks to complete the feature extraction, regression and classification operations of wood images. The YOLO, SSD and Densenet-SSD algorithms studied in this article belong to this category. This study compared the trained model with the above algorithms. The results of the comparison are shown in Table 7.

A comparison of the performance of other target detection algorithms in wood defect detection (Table 7), shows that our method performed well overall. Compared with the worst-performing mathematical morphology and ResNet152, the precision of the model proposed in this paper was improved by 12.7% to 96.1%, which met the precision requirement of not less than 95% of wood processing enterprises. The poor performance of the first method may be due to the different image quality, and the poor generalization of the mathematical morphology method in feature positioning. Compared with the two-step target detection algorithm Faster-RCNN, the one-step target detection algorithm SSD and YOLO-tiny can detect images faster, but the detection precision of traditional SSD algorithms is relatively low. The DenseNet-SSD algorithm we proposed improves the detection precision to 96.1% from 91.2% for the SSD algorithm, which is higher than the other methods. Although the detection time increases slightly, it is still far lower than other algorithms. The result can be explained by the fully connected layers and the dense connection between the convolutional layers. The dense connection feature means that the image information of each layer of the feature maps convolved to retain more image information than ResNet and VGG16, thus it has the advantage of network prediction to obtain accurate results, and uses multi-layer feature fusion to make the network more adaptable to defect targets with variable sizes.

However, our model still has some limitations. From the experimental results, we can see that the recognition precision of our model for dead knots and checking defects is more than 98%, but the precision rate for live knot defects is still around 90%, and was not significantly improved. The reason may be that live defects are more similar to background features than dead knots and checking defects. In addition, from the perspective of the training process of the model, the performance of the model is closely related to its defect location precision. The higher the location precision, the greater the classification precision.

## 5. Conclusions

Over time, many non-destructive wood testing theories and methods have emerged and the testing accuracy and testing speed have been greatly improved; however, it is often still necessary to sacrifice one of these to improve the other. Here, we adopted the transfer learning method to apply the traditional SSD algorithm to solid wood board defect recognition, and the SGD method and the Adam optimizer applied to the solid wood board defect data were compared in the training performance of the traditional SSD algorithm to obtain a better loss function optimizer. It was found that compared with SGD method, the Adam optimizer training could get faster loss value and lower loss value of 0.901, and the detection time was also reduced by 13 ms. However, because the VGG network is shallow and multiple convolution pooling can easily cause the loss of a lot of feature information, which causes gradient disappearance, the performance of traditional SSD networks in identifying defects in solid wood panels was unsatisfactory. To improve this situation and the recognition accuracy of solid wood board defects, the DenseNet121 network was introduced to replace the VGG16 network in the traditional SSD algorithm, and the idea of residual learning was adopted to increase the depth of the network while avoiding the loss of feature map information. After experimental verification, the detection performance of the solid wood board defect detection network improved, the mean average precision value increased by 4.6% to 96.1%, and the detection time only increased by about 38 ms, both of which satisfy the needs of wood processing enterprises. Also, the P-R curve corresponding to various defects was steeper, and the area under the curve also increased in AP value. The recall and precision rates of the model achieved a better balancing effect than those of traditional SSD algorithms. The research results have been applied recently in the wood sawing production line of Jiangjia Machinery Co., Ltd. in Jiangsu Province, China.

However, due to the limitations mentioned above, in the future, we need to explore the reasons why the precision rate of live joint defects has not been improved and other methods to improve this, and extend the model to the identification of other wood surface defects such as wormhole, discoloration and so on. In addition, we plan to study the regression method of target location information in target detection to improve the positioning precision of the target, thereby improving the overall precision of the target recognition.

## Figures and Tables

**Table 1:** Distribution of the Number of Defect Labels in the Data Sets.

Defect	Original Labels Data Set	Train Labels Data Set	Augmented Train Labels Data Set	Test Labels Data Set
Live knot	491	342	1026	149
Dead knot	229	159	477	70
Checking	194	136	408	58
Total	914	637	1911	277

**Table 2:** Software and Hardware Environment Configurations.

	Parameter
System	Windows 10 × 64
CPU	Inter Xeon W-2155@3.30GHz
GPU	Nvidia GeForce GTX 1080 Ti(11G)
Environment configuration	PyCharm + Pytorch1.2.0 + Python3.7.7
	Cuda10.0+cudnn7.6+tensorboardX2.1.0

**Table 3:** Surface Defect Detection Results of Solid Wood Panels Based on the SSD Algorithm.

Optimizer	Defect	Average Precision	Mean Average Precision	Mean Detect Time
SGD (moment = 0.9)	Live knot	89.7 ± 0.5%	90.4 ± 0.5%	30 ± 1 ms
	Dead knot	90.9 ± 0.5%		
	Checking	90.7 ± 1%		
Adam (betas = [0.9,0.99])	Live knot	90.2 ± 0.5%	91.2 ± 0.5%	17 ± 1 ms
	Dead knot	90.1 ± 0.5%		
	Checking	93.4 ± 0.5%		

**Table 4:** The VGGNet Structure.

ConvNet Configuration					
A	A-LRN	B	C	D	E
11 weight layers	11 weight layers	13 weight layers	16 weight layers	16 weight layers	19 weight layers
input (224 × 224 RGB image)					
conv3-64	conv3-64 LRN	conv3-64 conv3-64	conv3-64 conv3-64	conv3-64 conv3-64	conv3-64 conv3-64
maxpool					
conv3-128	conv3-128	conv3-128 conv3-128	conv3-128 conv3-128	conv3-128 conv3-128	conv3-128 conv3-128
maxpool					
conv3-256 conv3-256	conv3-256 conv3-256	conv3-256 conv3-256	conv3-256 conv3-256 conv1-256	conv3-256 conv3-256 conv3-256	conv3-256 conv3-256 conv3-256 conv3-256
maxpool					
conv3-512 conv3-512	conv3-512 conv3-512	conv3-512 conv3-512	conv3-512 conv3-512 conv1-512	conv3-512 conv3-512 conv3-512	conv3-512 conv3-512 conv3-512 conv3-512
maxpool					
conv3-512 conv3-512	conv3-512 conv3-512	conv3-512 conv3-512	conv3-512 conv3-512 conv1-512	conv3-512 conv3-512 conv3-512	conv3-512 conv3-512 conv3-512 conv3-512
maxpool					
FC-4096					
FC-4096					
FC-1000					
Soft-max					

**Table 5:** DenseNet Network Structure in the Improved SSD Algorithm for Solid Wood Board Defect Recognition.

Layers	DenseNet121 (k = 32)	Output Size	Channels
Input		200 × 200	3
Convolution	7 × 7 conv, stride 2	100 × 100	64
Pooling	3 × 3 max pool, stride 2	50 × 50	64
Dense Block (1)	Bottleneck × 6	50 × 50	256
Transition Layer (1)	1 × 1 conv	50 × 50	128
	2 × 2 average pool, stride 2	25 × 25	128
Dense Block (2)	Bottleneck × 12	25 × 25	512
Transition Layer (2)	1 × 1 conv	25 × 25	256
	2 × 2 average pool, stride 2	12 × 12	256
Dense Block (3)	Bottleneck × 24	12 × 12	1024
Transition Layer (3)	1 × 1 conv	12 × 12	512
	2 × 2 average pool, stride 2	6 × 6	512
Dense Block (4)	Bottleneck × 16	6 × 6	1024
Conv Layer	1 × 1 conv	6 × 6	1024

**Table 6:** Experimental Results of DenseNet-SSD in Solid Wood Board Defect Detection.

Network	Defect	True Positive	False Positive	Recall	Average Precision	Mean Average Precision	Mean Detect Time
DenseNet-SSD	Live knot	147	44	98.7%	90.5 ± 0.3%	96.1 ± 0.3%	56 ± 1 ms
	Dead knot	70	8	100%	98.6 ± 0.3%		
	Checking	58	27	100%	99.0 ± 0.3%		

**Table 7:** Comparison of Different Algorithms.

Algorithms	Mean Precision (%)	Time (ms)
mathematical morphology + ResNet152	83.4	1012
Faster-RCNN	93	870
YOLO-tiny	95.2	152
SSD	91.2	17
DenseNet-SSD	96.1	56

## References

[1] Fan J., Liu Y., Hu Z., Zhao Q., Shen L., Zhou X. (2019). Solid wood panel defect detection and recognition system based on Faster R-CNN. J. For. Eng..

[2] Anderson G.C., Owens F.C., Franca F., Ross R.J., Shmulsky R. (2020). Correlations between Grain Angle Meter Readings and Bending Properties of Mill-Run Southern Pine Lumber. Forest Prod. J..

[3] Kim K. (2007). A note on the Hankinson formula. Wood Fiber Sci..

[4] Cao J., Liang H., Lin X., Tu W., Zhang Y. (2016). Potential of Near-infrared Spectroscopy to Detect Defects on the Surface of Solid Wood Boards. Bioresources.

[5] Thumm A., Riddell M. (2017). Resin defect detection in appearance lumber using 2D NIR spectroscopy. Eur. J. Wood Prod..

[6] Yu H., Liang Y., Liang H., Zhang Y. (2019). Recognition of wood surface defects with near infrared spectroscopy and machine vision. J. For. Res..

[7] Mousavi M., Taskhiri M.S., Holloway D., Olivier J., Turner P. (2020). Feature extraction of wood-hole defects using empirical mode decomposition of ultrasonic signals. NDT E Int..

[8] Taskhiri M.S., Hafezi M.H., Harle R., Williams D., Kundu T., Turner P. (2020). Ultrasonic and thermal testing to non-destructively identify internal defects in plantation eucalypts. Comput. Electron. Agric..

[9] Yang H., Yu L. (2016). Feature extraction of wood-hole defects using wavelet-based ultrasonic testing. J. For. Res..

[10] Wang Q.P., Liu X., Yang S.M. (2020). Predicting Density and Moisture Content of Populus xiangchengensis and Phyllostachys edulis using the X-Ray Computed Tomography Technique. For. Prod. J..

[11] Rummukainen H., Makkonen M., Uusitalo J. (2019). Economic value of optical and X-ray CT scanning in bucking of Scots pine. Wood Mater. Sci. Eng..

[12] Qin R., Qiu Q., Lam J.H.M., Tang A.M.C., Leung M.W.K., Lau D. (2018). Health assessment of tree trunk by using acoustic-laser technique and sonic tomography. Wood Sci. Technol..

[13] Qiu Q.W., Lau D. (2019). Grain effect on the accuracy of defect detection in wood structure by using acoustic-laser technique. Proc. SPIE.

[14] Campbell L., Edwards K., LeMaster R., Velarde G. (2018). The Use of Acoustic Emission to Detect Fines for Wood-Based Composites, Part One: Experimental Setup for Use on Particleboard. Bioresources.

[15] Li X.C., Li M., Ju S. (2020). Frequency Domain Identification of Acoustic Emission Events of Wood Fracture and Variable Moisture Content. For. Prod. J..

[16] Nasir V., Nourian S., Avramidis S., Cool J. (2019). Stress wave evaluation for predicting the properties of thermally modified wood using neuro-fuzzy and neural network modeling. Holzforschung.

[17] Ni C., Li Z., Zhang X., Sun X., Huang Y., Zhao L., Zhu T., Wang D. (2020). Online Sorting of the Film on Cotton Based on Deep Learning and Hyperspectral Imaging. IEEE Access.

[18] He T., Liu Y., Xu C., Zhou X., Hu Z., Fan J. (2019). A Fully Convolutional Neural Network for Wood Defect Location and Identification. IEEE Access.

[19] Hu K., Wang B.J., Shen Y., Guan J.R., Cai Y. (2020). Defect Identification Method for Poplar Veneer Based on Progressive Growing Generated Adversarial Network and MASK R-CNN Model. Bioresources.

[20] Shi J., Li Z., Zhu T., Wang D., Ni C. (2020). Defect Detection of Industry Wood Veneer Based on NAS and Multi-Channel Mask R-CNN. Sensors.

[21] Kurdthongmee W., Suwannarat K. Locating Wood Pith in a Wood Stem Cross Sectional Image Using YOLO Object Detection. Proceedings of the 2019 International Conference on Technologies and Applications of Artificial Intelligence (TAAI).

[22] Franca F.J.N., Franca T.S.F.A., Seale R.D., Shmulsky R. (2020). Nondestructive Evaluation of 2 by 8 and 2 by 10 Southern Pine Dimensional Lumber. Forest Prod. J..

[23] He K., Zhang X., Ren S., Sun J. Deep Residual Learning for Image Recognition. Proceedings of the 2016 IEEE Conference on Computer Vision and Pattern Recognition (CVPR).

[24] Girshick R., Donahue J., Darrell T., Malik J., Malik J. Rich Feature Hierarchies for Accurate Object Detection and Semantic Segmentation. Proceedings of the 2014 IEEE Conference on Computer Vision and Pattern Recognition.

[25] Girshick R. Fast R-CNN. Proceedings of the 2015 IEEE International Conference on Computer Vision (ICCV).

[26] Ren S.Q., He K.M., Girshick R., Sun J. (2017). Faster R-CNN: Towards Real-Time Object Detection with Region Proposal Networks. IEEE T Pattern Anal.

[27] Liu W., Anguelov D., Erhan D., Szegedy C., Reed S., Fu C.Y., Berg A.C. SSD: Single Shot MultiBox Detector. Proceedings of the the 14th European Conference on Computer Vision.

[28] Redmon J., Divvala S., Girshick R., Farhadi A. You Only Look Once: Unified, Real-Time Object Detection. Proceedings of the 2016 IEEE Conference on Computer Vision and Pattern Recognition (CVPR).

[29] Neubeck A., Van Gool L. Efficient Non-Maximum Suppression. Proceedings of the 18th International Conference on Pattern Recognition (ICPR’06).

[30] Huang G., Liu Z., Van Der Maaten L., Weinberger K.Q. Densely Connected Convolutional Networks. Proceedings of the 2017 IEEE Conference on Computer Vision and Pattern Recognition (CVPR).

[31] Kamal K., Qayyum R., Mathavan S., Zafar T. (2017). Wood defects classification using laws texture energy measures and supervised learning approach. Adv. Eng. Informatics.

[32] Xiao J.Y., Ma Y., Zhou Z., Fang Y.M. (2020). Detection of Timber Surface Knots Based on Faster R-CNN. China Wood Ind..

[33] Urbonas A., Raudonis V., Maskeliunas R., Damaševičius R. (2019). Automated Identification of Wood Veneer Surface Defects Using Faster Region-Based Convolutional Neural Network with Data Augmentation and Transfer Learning. Appl. Sci..

